# Mental Health Outcomes Among Long‐Term Survivors of Childhood, Adolescent and Young Adult Cancer: A Scottish Population‐Based Cohort Study

**DOI:** 10.1002/pon.70529

**Published:** 2026-06-21

**Authors:** Emanuela Molinari, Colin McLean, Ines Mesa‐Eguiagaray, Sarah H. Wild, Peter S. Hall

**Affiliations:** ^1^ School of Informatics University of Edinburgh Edinburgh UK; ^2^ Institute of Genetics and Cancer University of Edinburgh Edinburgh UK; ^3^ Department of Psychology Lothian Birth Cohorts The University of Edinburgh Edinburgh UK; ^4^ Usher Institute College of Medicine and Veterinary Medicine University of Edinburgh Edinburgh UK

**Keywords:** adolescent, cancer, cohort studies, mental health, oncology, primary health care, psychotropic drugs, survivors, survivorship, young adult

## Abstract

**Background:**

Survivors of cancer face increased risks of long‐term morbidity compared to people without a cancer history.

**Aims:** To quantify the long‐term burden of mental health morbidity and to evaluate the healthcare settings in which these events are identified in long‐term Scottish cancer survivors diagnosed before 40 years of age.

**Methods:**

Five‐year survivors of cancer diagnosed before 40 years of age in the Lothian region of Scotland between 1980 and 2018 (*n* = 8862) were matched (1:3) to individuals without cancer history by age, sex, and socioeconomic deprivation (*n* = 26,586). Mental‐health outcomes were identified from psychotropic drug prescribing, hospital admission and general‐practice records. Time to first mental health event was analysed using time‐stratified Cox proportional‐hazards models.

**Results:**

During median follow‐up of 14.8 years for survivors and 16.7 years for comparators, 2983 survivors (33.7%) and 6774 comparators (25.5%) experienced a first mental health event. Around 90% of first events were identified in general practice or prescribing records, and fewer than 10% in hospital admissions. Survivors had a consistently higher incidence of first mental health events across follow‐up intervals when all data sources were considered, with hazard ratios ranging from 1.3 in the first year after study entry (95% CI 1.2–1.4) to 1.5 beyond 15 years (95% CI 1.4–1.6).

**Conclusions:**

Integrating data sources provides a more comprehensive assessment of the mental health burden than hospital records alone. Mental health should be a core component of long‐term survivorship care, particularly within primary care, and cost‐effective strategies are needed to address the sustained excess burden.

## 1 Background

Mental health is a fundamental component of overall well‐being [1] and is particularly relevant for survivors of childhood, adolescent, and young adult (CAYA) cancer survivors, who endure both the physical and psychological consequences of their illness and treatment during critical periods of emotional and social development [2, 3, 4].

There is increasing recognition of the need to describe long‐term outcomes in CAYA cancer survivors [5, 6, 7]. However, evidence on mental health remains limited [4, 8] and often relies on single data sources that may underestimate the true burden or fail to capture the full spectrum of mental health care needs.

Previous studies using hospital records have reported increased risks of psychiatric hospital admissions [9, 10, 11] among long‐term survivors of CAYA cancers. Studies based on prescribing data have similarly reported an increased use of psychotropic medications in this population [12, 13, 14], suggesting a higher burden of mental health conditions managed in outpatient settings. In contrast, Scottish registry‐based analyses based solely on hospital admissions found that the risk of psychiatric hospital admission in survivors of cancer diagnosed under 25 years of age between 1981 and 2003 was similar to that of the general population (standardised hospitalisation ratio 0.9; 95% CI 0.8–1.2) [15]. Clarifying whether survivors are more vulnerable to developing mental health morbidity is important, as mental health disorders in the general population have been found to be associated with reduced life expectancy [15] and with increased suicide risk among children cancer survivors [16].

Scotland now offers a unique opportunity to investigate the conflicting findings and address some of the limitations of previous studies through comprehensive data linkage of routinely collected healthcare data. The use of the unique patient identifier, the Community Health Index (CHI), enables integration of cancer registry and mortality data with primary care, secondary care and medication dispensing data. Using these linked data, this study describes and quantifies the long‐term mental health burden across the spectrum of care among individuals who survived at least 5 years after a cancer diagnosis before 40 years of age and examines whether outcome definition and data source influence estimates of mental health morbidity.

## 2 Methods

### 2.1 Study Design and Population

We conducted a retrospective matched cohort study using routinely collected health data accessed through the DataLoch Trusted Research Environment, a secure Safe Haven that enables linkage of regional secondary care and prescribing datasets with primary care records. Individuals residing in the Lothian region who were diagnosed with invasive cancer through the Scottish Cancer Registry (SMR06) before 40 years of age (see Supporting Information Table S1) who survived at least 5 years from cancer diagnosis between 1 January 1985 and 31 December 2018 (*n* = 8862). The cohort size was determined by the number of eligible individuals available within the extracted datasets for the study period. Each survivor was matched to three cancer‐free comparators (*n* = 26,586) on year of birth, sex, and socioeconomic status defined by quintiles of the Scottish Index of Multiple Deprivation (SIMD, version 2020v2).

### 2.2 Data Sources and Linkage

Linked datasets included the Scottish Morbidity Records (SMR) for general (SMR01) and psychiatric admissions (SMR04), general practice (GP) records, the cancer registry (SMR06), community prescribing information system (PIS) of dispensed prescriptions, and death records (from the National Records of Scotland, NRS). Records were provided to the analytic platform in de‐identified form.

### 2.3 Mental Health Event Identification

Mental health events were identified using multiple data sources, including hospital discharge diagnoses (SMR01/SMR04), general practice records, and psychotropic medication dispensing from the Prescribing Information System. The first mental health event was defined as the earliest record across any source. Dispensed prescriptions were considered indicators of mental health events when consistent with sustained treatment (dispensed for more than 6 months) and after applying criteria to minimise misclassification for non‐psychiatric indications, such as pain, migraine, epilepsy, dementia. Detailed diagnostic codes and prescribing definitions are provided in Supplementary Material (Supporting Information Table S2, Figure S1). Individuals with any mental health record across data sources occurring before cancer diagnosis (or pseudo‐diagnosis for comparators) were classified as having prior mental health history. Mental health events occurring between diagnosis and cohort entry (5 years post‐diagnosis) were not included.

### 2.4 Main Outcome and Covariates

The primary outcome was time to first mental health event. Models accounted for age at diagnosis or pseudo‐date for comparators (0–9, 10–24, 25–39 years) and decade of diagnosis. These variables accounted for key developmental periods and changes in treatment regimens and clinical practice over time. Sex and prior mental health history were used as stratification variables. Socioeconomic status was not included in models as it was a matching variable. Covariate selection was guided by a priori clinical and epidemiological knowledge to account for potential confounding while avoiding adjustment for variables on the causal pathway.

### 2.5 Statistical Analysis

Descriptive statistics were used to summarise demographic and clinical characteristics for survivors and matched comparators. Continuous variables were not normally distributed; therefore, they were summarised using medians and interquartile ranges (IQRs), while categorical variables were expressed as counts and percentages.

Person‐years at risk were calculated from the cohort entry date (5 years after cancer diagnosis date for survivors and their matched comparators) and ended at the first mental health event, death, or 1 October 2024, whichever occurred first. Subsequent cancer diagnoses occurring during follow‐up, including second primary cancers, were not treated as new index events, and follow‐up continued for such individuals.

Cox proportional‐hazards models estimated hazard ratios for survivors versus comparators. Analyses were conducted using complete‐case data. Model assumptions were evaluated using standard diagnostic methods (see Supplementary Methods). The proportional hazards assumption was assessed using Schoenfeld residuals. Time‐stratified analyses were conducted where non‐proportionality was observed after exploration of Kaplan–Meier curves (not accounting for competing risks) and number of events in each time interval. Cox models were then fitted across four predefined intervals: 0–1, > 1–5, > 5–10, > 10–15, and > 15 years since cohort entry. The proportional‐hazards assumption was checked within each interval. Hazard ratios (HRs) with 95% confidence intervals (CIs) were reported for the first mental health event. Statistical significance was defined as *p* < 0.05.

### 2.6 Sensitivity Analyses

Sensitivity analyses restricted outcome ascertainment to hospital records only (SMR01 and SMR04) and to the period with complete primary care and prescribing data (from 2010 onwards, with cancer cohort diagnosis from 2005 to 2018).

### 2.7 Statistical Environment, Software, and Packages

Statistical analyses were conducted within the secure DataLoch analytical environment. Details of the statistical software and packages used are provided in Supplementary Material (Supplementary Methods).

## 3 Ethical Approval

Ethical approval was granted by the NHS Research Ethics Committee (REC) (DL‐2021–045). The requirement for individual informed consent was waived by the REC because the study used de‐identified routinely collected health data. Data were accessed through the secure DataLoch Trusted Research Environment (TRE) in compliance with the General Data Protection Regulation (GDPR) and institutional data governance policies.

## 4 Results

### 4.1 Cohort Characteristics

The final cohort included 8862 cancer survivors and 26,586 matched comparators. Individuals with incomplete follow‐up information (*n* = 3) and their matched comparators were excluded. Baseline demographic characteristics were similar between groups and are summarised in Table 1, with 5‐year survivor characteristics provided in Supplementary material (Supporting Information S1: Table S3). The cohort was predominantly female (60%), reflecting the impact of breast and gonadal tumours in this age group, and the distribution of socioeconomic deprivation was comparable between groups.

**TABLE 1:** Demographic and clinical characteristics of the cohort of survivors of cancer diagnosed < 40 years of age in the Lothian region of Scotland 1980–2013 and comparators population.

Characteristic	Survivors (*n* = 8862)	Comparators (*n* = 26,586)
Sex, *n* (*n*, %)		
Female	5361 (60.5)	16,190 (60.9)
Male	3501 (39.5)	10,396 (39.1)
SIMD quintile, *n* (%)		
1 (most deprived)	1207 (13.6)	3114 (11.7)
2	1888 (21.3)	5636 (21.2)
3	1626 (18.3)	5181 (19.5)
4	1609 (18.2)	4847 (18.2)
5 (least deprived)	2532 (28.6)	7808 (29.4)
Prior mental health history, *n* (%)	78 (0.9)	145 (0.5)
Age (median, IQR)	32.1 (25.1–36.6)	32.4 (25.3–36.7)

*Note:* Percentages are column percentages unless otherwise stated.

Abbreviations: SIMD, Scottish Index of Multiple Deprivation; IQR, interquartile range.

Median follow‐up from cohort entry (5‐year post‐diagnosis) was 12.1 years (IQR 4.4–22.0) for survivors and 13.4 years (IQR 5.7–22.8) for comparators, contributing to totals of 110,646 and 393,203 person‐years of follow‐up respectively; the greater person‐time among comparators reflects the 1:3 matching ratio.

During the study period, 1369 (15.5%) of survivors and 1682 (6.1%) of comparators died after cohort entry. Median age at death was 48.2 years among survivors and 59.9 years among comparators.

### 4.2 Mental Health Outcome

A total of 2983 (33.7%) survivors and 6774 (25.5%) comparators experienced a new mental health event during follow‐up (Supporting Information Figure S1).

Among participants with a first mental health event after cohort entry 275 survivors (9.2%) and 616 comparators (9.1%) had a psychiatric diagnosis recorded in general hospital admissions and 31 survivors (1.0%) and 64 comparators (0.9%) had a record of psychiatric hospital admission. GP data provided records of first mental health event for 1682 survivors (56.4%) and 4145 comparators (61.2%) and community prescribing (PIS) provided evidence of first mental health events for 995 survivors (33.4%) and 1949 comparators (28.8%). When prescribing data was cross‐referenced with GP diagnostic coding, a total of 4745 prescriptions were dispensed for indications unlikely to reflect mental health morbidity, representing 25.3% of all first psychotropic prescriptions. Nearly half of all first antidepressant prescriptions were issued in the context of non‐mental health use such as pain or migraine (46.5% among survivors, *n* = 1126), 43.3% among comparators, *n* = 2946). Antipsychotic prescriptions that were accompanied by a dementia diagnosis accounted for 6.7% of survivors (*n* = 29) and 10.2% of comparators (*n* = 64). No alternative psychiatric indications were detected for medications used for substance dependence, anxiolytics, or hypnotics.

### 4.3 Main Analysis

Initial Cox proportional‐hazards models assessing the association between survivor status and risk of a first mental health event indicated violation of the proportional hazards assumption. Schoenfeld residuals demonstrated significant time‐dependency for both the cohort effect and sex. Kaplan‐Meier curves (Supporting Information Figure S2) visualisation suggested that hazard differences between survivors and comparators were not constant over time, with the largest divergence observed within the first decade of follow‐up (log‐rank test *p* < 0.001). Piecewise Cox models were implemented, and the same Cox model was applied to the five predefined intervals: 0–1, > 1–5, > 5–10, > 10–15, and > 15 years since cohort entry which held proportional hazards assumption. Hazard ratios ranged from 1.3 (95% CI 1.2–1.4) in the early interval to 1.5 (95% CI 1.4–1.6) at later time points, indicating a persistent excess risk throughout long‐term follow‐up (Table 2). Proportional hazards assumptions were satisfied. Age at diagnosis and decade of diagnosis had limited influence on subsequent mental health morbidity (Supporting Information Table S4).

**TABLE 2:** Time‐stratified cox proportional‐hazards models for first mental health event among 5‐year cancer survivors and matched comparators.

Follow‐up interval (years)	Events Survivors	Events Comparators	Hazard ratio (HR)	95% CI	*p*‐value
0–1	772	1241	1.11	1.01–1.22	0.03
> 1–5	741	1580	1.33	1.20–1.47	< 0.001
> 5–10	538	1365	1.38	1.23–1.54	< 0.001
> 10–15	410	1052	1.42	1.25–1.62	< 0.001
> 15	522	1536	1.44	1.29–1.61	< 0.001

*Note:* HRs were estimated using time‐stratified Cox proportional hazards models comparing survivors with matched comparators. Models were stratified by sex and prior mental health history and adjusted for age group and decade of diagnosis. Events indicate the number of first mental health events in each interval.

### 4.4 Sensitivity Analysis

**FIGURE 1:**
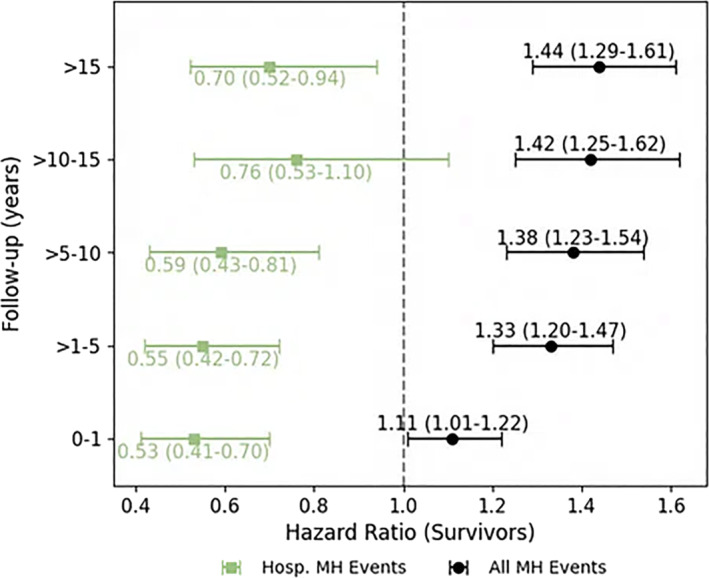
Comparison of hazard ratios (HRs) for survivors versus comparators across follow‐up intervals for all mental health outcomes and outcomes limited to those identified from general and psychiatric hospital admissions. HRs between the composite outcome (black) and hospital‐only outcome (green) across follow‐up intervals. The figure shows that the composite outcome yielded consistently elevated risks for survivors, whereas hospital‐only outcomes indicated lower relative hazards that attenuated over time, consistent with the null findings reported by Brewster et al. (2014). Shaded bands represent 95% confidence intervals.

When mental health morbidity was restricted to hospital records of mental health diagnosis or psychiatric hospital admission only (SMR01 and SMR04), survivors showed lower hazards of mental health events compared with matched comparators, with hazard ratios below unity across all follow‐up intervals (ranging from 0.53 [95% CI 0.41–0.70] in the first year to 0.76 [95% CI 0.53–1.10] after 10–15 years) (Figure 1).

**FIGURE 2:**
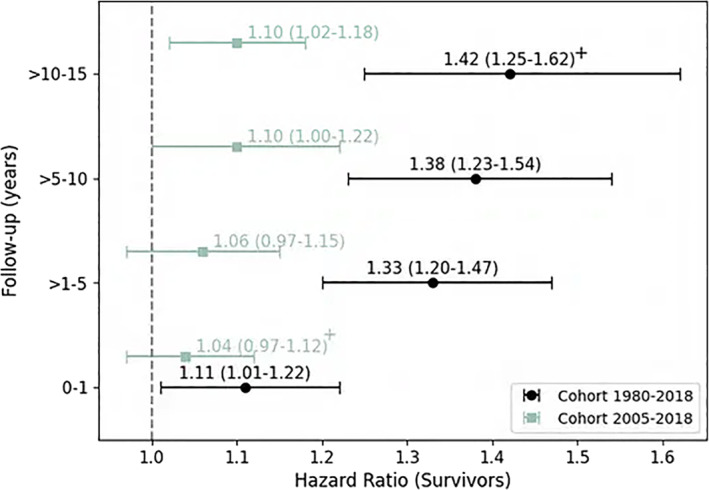
Comparison of hazard ratios (HRs) between the full cohort versus all data access. HRs for survivors and comparators across the full cohort (black) versus when limited to cases diagnosed between 2005 and 2018 (green), with full data coverage across all data sources. The follow up is shorter due to later diagnosis years. In this restricted cohort, survivors continued to show a significantly higher incidence of mental health events throughout follow‐up, confirming the robustness of results to differences in data‐source completeness. Intervals marked with (+) indicate mild deviation from the proportional‐hazards assumption.

When the analysis was restricted to individuals diagnosed between 2005 and 2018, corresponding to the period of complete primary‐care and prescribing data coverage, follow‐up duration was shorter than in the main cohort, limiting the assessment of long‐term outcomes. Survivors had a significantly higher hazards of mental health morbidity than comparators over the whole follow‐up period (HR 1.34, 95% CI 1.23–1.46; *p* < 0.001) (Figure 2), similar to the findings of the main analysis. Proportional hazards assumptions remained satisfied across time intervals.

## 5 Discussion

In this population‐based retrospective matched cohort study, we found that individuals diagnosed with cancer before 40 years of age who survived at least 5 years had a higher incidence of first mental health events compared with age‐, sex‐, deprivation‐matched comparators with no history of cancer diagnosed under 40 years of age. The risk of developing a new mental health event increased over time, increasing from approximately 30% in the early years of long‐term survivorship to around 40%–50% beyond 15 years of follow up. The relative increase in risk observed in this study is broadly consistent with previous reports. However, by incorporating primary care and prescribing data in addition to hospital records, we identified substantially higher absolute rates of mental health events than studies based on hospital data alone. Because additional events were captured in both survivors and comparators, the relative risk remained similar, while the proportion of individuals affected was considerably greater. These findings indicate that hospital‐based studies substantially underestimate the overall burden of mental health morbidity in cancer survivorship and highlight the importance of considering both relative and absolute risk when assessing survivorship needs.

This study is, to our knowledge, among the first UK studies to integrate primary care, secondary care, and prescribing data into a comprehensive measure of mental health burden in young cancer survivors. The cohort is also more contemporary than most prior population‐based studies, reflecting modern treatment and survivorship patterns and improving the relevance of the findings to current clinical practice. In addition, our study includes young adults aged 18–39 years, a group underrepresented in previous research despite distinct developmental and psychosocial challenges.

Our findings are consistent with population‐based register cohorts showing higher rates of clinically recorded psychiatric morbidity in survivors of CAYA cancers [17] and when broader endpoints that included psychotropic prescriptions [12, 13, 14], outpatients data [10, 11, 18] and primary care data [10, 19] were used. By restricting our analysis to psychiatric diagnoses from hospital data alone, we also confirmed that hospitalisations capture only a small proportion of mental health morbidity among long‐term survivors. We found no significant association between survivorship status and hospital admissions for mental health diagnosis either in general acute or psychiatric hospitals. This result was consistent with a previous Scottish hospital‐based study of survivors diagnosed before 25 years of age compared to the general population [20]. These findings suggest that the previously reported null associations may reflect under‐ascertainment of mental health morbidity, rather than an absence of excess mental health burden among cancer survivors.

It remains to be determined why Scottish hospital data differed from those of other countries studying similar age groups, but potential explanations include differences in data sources, admission thresholds, coding practices, and the management of mental health conditions within primary care.

The simultaneous integration of multiple data sources facilitated a broader capture of mental health events, and addressed some of the key methodological challenges common in survivorship research, achieving a more complete ascertainment of clinically recorded mental health events than approaches based solely on hospital data that focus on severe mental health presentations. Linking prescriptions with underlying clinical diagnoses improved the specificity of outcome classification and minimised the inherent limitation of prescribing alone. The matched cohort design and stratification by prior mental health history, allowed us to estimate the excess incidence of new‐onset mental health events associated with cancer diagnosed at a young age.

Importantly, we demonstrated that the majority of first mental health presentations occurred in primary care settings (around 90% of first events). Linking primary care and prescribing data improved identification of mental health outcomes.

### 5.1 Study Limitations

This study has several limitations. The cohort was limited to the Lothian region of Scotland, a relatively affluent region, which may not reflect mental health care access or cancer survivorship patterns in more remote, rural, or more socio‐economically deprived areas in Scotland or in other countries. Accordingly, the distribution of socioeconomic deprivation quintiles in the study reflects the underlying population structure of the region, with a higher proportion of individuals in the least deprived categories than in Scotland as a whole. However, as a consequence of matching the socioeconomic distribution was similar between survivors and matched comparators, with representation across the deprivation spectrum (Table 1). The socio‐economic deprivation status was based on the last recorded postcode per dataset, which may reflect post‐diagnosis social change, therefore lying on the causal pathway between cancer survivorship and later social and economic outcomes, potentially acting as a mediator rather than a confounder. Linkage across care settings, enabling more complete ascertainment of mental health outcomes than would be possible using national datasets alone, was only feasible in the Lothian region of Scotland. Although this cohort is likely to be more representative than hospital‐based or tertiary‐centre study populations, findings may be most applicable to publicly funded healthcare systems with similar access pathways to mental health care.

Another limitation concerned the use of data sources with different start dates, potentially leading to incomplete event capture in earlier years. However, this is unlikely to have biased the comparison between survivors and controls, since matching was based on age at diagnosis (or pseudo‐diagnosis for comparators) and year of birth where possible. Although the onset of a mental health episode might have been delayed in the records of older cohorts, the inclusion of both primary care and prescribing data increased the likelihood that clinically significant mental health outcomes were captured. Our sensitivity analysis restricted to the recent cohort (study entry in 2010) for which all data sources were available, confirmed that the observed excess morbidity among survivors was unlikely to be explained by differences in data availability alone.

Linkage of primary care, prescribing, and hospital data enabled broader ascertainment of mental health outcomes across care settings. In the National Health Service (NHS), individuals generally first present to primary care, where symptoms may be recorded or referrals made prior to accessing private services. Privately delivered mental health care is not captured in routine NHS‐linked datasets, and some under‐ascertainment remains possible.

Prescribing patterns are useful indicators but have limited interpretability when used as proxies for health outcomes. In our cohort, 12.8% of dispensed drugs of interest were likely to have been prescribed for non‐mental health conditions, including pain, seizures, and dementia, but dual‐purpose prescribing may still have occurred, particularly where mental health diagnoses were clinically relevant but not formally coded. Consequently, some mental health indications may have been under‐recorded if they were not explicitly documented alongside the primary condition. Due to sample size constraints and missing treatment data (nearly one‐third of survivors lacked treatment information in SMR06), granular analyses by tumour type, age at diagnosis, and mental health category were not feasible, and inferring treatment variables may have introduced bias.

As our primary analyses used standard time‐to‐event methods treating death as a censoring event, estimates of time to first mental health event may be affected if death was informative (i.e., if individuals at higher risk of mental health events were also at higher risk of death). However, deaths were less frequent than mental health events, and the persistent excess incidence observed across follow‐up suggests that competing mortality is unlikely to fully explain the association. Future studies using competing‐risk methods would provide a more complete assessment of the impact of differential mortality.

Similarly, reverse causality due to deterioration in physical health cannot be excluded. Within our data, cancer recurrence could not be reliably distinguished from second primary cancers through registry coding. Tumour recurrence, second primary cancers, and treatment‐related comorbidities form part of the broader survivorship trajectory and may have complex, multifactorial and bidirectional relationships with mental health outcomes. We identified an overall association between cancer survivorship and incident mental health outcomes; further studies with longitudinal and more granular data are needed to investigate the temporal relationships of outcomes of interest.

### 5.2 Clinical Implications

Long‐term survivors of young‐onset cancer experience a sustained excess risk of mental health morbidity extending well beyond the initial survivorship period. As most first presentations were identified in primary care, general practitioners play a central role in the detection and management of mental health presentations in this population. Survivorship care pathways should therefore incorporate long‐term mental health surveillance, proactive screening to identify vulnerable individuals, and timely access to psychological and psychosocial support services.

### 5.3 Implications for Research

Future research should explore mental health events beyond the first occurrence, their recurrence patterns, and their impact on life expectancy and quality of life among survivors. Such studies would further clarify the persistence and cumulative burden of long‐term mental health morbidity following early‐age cancer diagnosis and survivorship and potentially identify high‐risk subgroups. If these findings are confirmed in larger or national cohorts, the effectiveness and cost‐effectiveness of targeted screening and intervention strategies within survivorship care should be evaluated.

## 6 Conclusions

Scottish young individuals who survive cancer for at least 5 years after diagnosis face a substantially higher risk of developing new mental health conditions compared with matched peers without cancer. The majority of mental health events are identified in primary care, underscoring the importance of integrated mental health surveillance across care settings. Linking primary care, hospital, and prescribing data provides a comprehensive and feasible approach for monitoring mental health outcomes in long‐term cancer survivors. This work represents the first step to design a surveillance system to support the evaluation of interventions aimed at reducing long‐term mental health morbidity in cancer survivorship.

**Abbreviations**
BNF: British National Formulary
CHI: Community Health Index
CI: Confidence Interval
CAYA: Childhood, Adolescent, and Young Adult
GP: General Practitioner
HRs: Hazard Ratios
ICD: International Classification of Diseases
NHS: National Health Service
NRS: National Records of Scotland
PH: Proportional Hazard
PIS: Prescribing Information System
SMR: Scottish Morbidity Record
UK: United Kingdom
WHO: World Health Organisation

## Author Contributions

**Emanuela Molinari:** conceptualization, data application and curation, funding acquisition, resources, data curation, methodology, formal analysis, visualization, interpretation, writing – original draft, review and editing. **Peter Hall:** data application, project administration, resources, methodology, supervision, interpretation, writing – original draft, review and editing. **Colin McLean:** software, project administration, supervision, writing – review and editing. **Ines Mesa‐Eguiagaray:** Statistical advice, methodology, review and editing. **Sarah Wild:** methodology, supervision, data interpretation, writing – review and editing.

## Funding

The authors have nothing to report.

## Conflicts of Interest

The authors declare no conflicts of interest.

## Supporting information


Supporting Information S1

## Data Availability

The data that support the findings of this study are available on request via DataLoch application. The data are not publicly available due to privacy or ethical restrictions.

## References

[1] D. Kestel , D. Chisholm , and A. Schafer , “The WHO Special Initiative for Mental Health: Increasing Access to Mental Health Services for Millions of People,” World Psychiatry 24, no. 1 (2025): 137–138, 10.1002/wps.21286. 39810689 PMC11733446

[2] A. S. E. Darlington , C. E. Wakefield , L. M. E. van Erp , W. T. A. van der Graaf , R. J. Cohn , and M. A. Grootenhuis , “Psychosocial Consequences of Surviving Cancer Diagnosed and Treated in Childhood Versus in Adolescence/Young Adulthood: A Call for Clearer Delineation Between Groups,” Cancer 128, no. 14 (2022): 2690–2694, 10.1002/cncr.34257. 35579570

[3] N. Itzep and M. Roth , “Psychosocial Distress due to Interference of Normal Developmental Milestones in Ayas With Cancer,” Children 9, no. 3 (2022): 309, 10.3390/children9030309. 35327680 PMC8947616

[4] A. R. Y. B. Lee , C. E. Low , C. E. Yau , J. Li , R. Ho , and C. S. H. Ho , “Lifetime Burden of Psychological Symptoms, Disorders, and Suicide due to Cancer in Childhood, Adolescent, and Young Adult Years: A Systematic Review and Meta‐Analysis,” JAMA Pediatrics 177, no. 8 (2023): 790–799, 10.1001/jamapediatrics.2023.2168. 37345504 PMC10288378

[5] W. H. Chang and A. G. Lai , “Cumulative Burden of Psychiatric Disorders and Self‐Harm Across 26 Adult Cancers,” Nature Medicine 28, no. 4 (2022): 860–870, 10.1038/s41591-022-01740-3. PMC9018408 35347280

[6] S. H. M. Janssen , W. T. A. van der Graaf , D. J. van der Meer , E. Manten‐Horst , and O. Husson , “Adolescent and Young Adult (Aya) Cancer Survivorship Practices: An Overview,” Cancers 13, no. 19 (2021): 4847, 10.3390/cancers13194847. 34638332 PMC8508173

[7] J. K. McLoone , U. M. Sansom‐Daly , A. Paglia , et al., “A Scoping Review Exploring Access to Survivorship Care for Childhood, Adolescent, and Young Adult Cancer Survivors: How Can We Optimize Care Pathways?,” Adolescent Health, Medicine and Therapeutics 14 (2023): 153–174, 10.2147/ahmt.s428215. 37753163 PMC10519427

[8] V. Osmani , L. Hörner , S. J. Klug , and L. F. Tanaka , “Prevalence and Risk of Psychological Distress, Anxiety and Depression in Adolescent and Young Adult (AYA) Cancer Survivors: A Systematic Review and Meta‐Analysis,” Cancer Medicine 12, no. 17 (2023): 18354–18367, 10.1002/cam4.6435. 37559504 PMC10523984

[9] L. Ross , C. Johansen , S. O. Dalton , et al., “Psychiatric Hospitalizations Among Survivors of Cancer in Childhood or Adolescence,” New England Journal of Medicine 349, no. 7 (2003): 650–657, 10.1056/nejmoa022672. 12917301

[10] P. C. Nathan , A. Nachman , R. Sutradhar , et al., “Adverse Mental Health Outcomes in a Population‐Based Cohort of Survivors of Childhood Cancer,” Cancer 124, no. 9 (2018): 2045–2057, 10.1002/cncr.31279. 29468664

[11] R. De , R. Sutradhar , P. Kurdyak , et al., “Incidence and Predictors of Mental Health Outcomes Among Survivors of Adolescent and Young Adult Cancer: A Population‐Based Study Using the IMPACT Cohort,” Journal of Clinical Oncology 39, no. 9 (2021): 1010–1019, 10.1200/jco.20.02019. 33492982

[12] Y. T. Cheung , W. Liu , T. M. Brinkman , et al., “Prescription Psychoactive Medication Use in Adolescent Survivors of Childhood Cancer and Association With Adult Functional Outcomes,” JNCI Cancer Spectrum 4, no. 5 (2020): pkaa057, 10.1093/jncics/pkaa057. 33134833 PMC7583158

[13] R. Montague , S. E. Canning , P. Thielking , and F. Qeadan , “Adverse Childhood Experiences and Psychotropic Medication Prescription Among Cancer Patients,” Journal of Psychosocial Oncology 42, no. 4 (2024): 543–557, 10.1080/07347332.2023.2296040. 38127059

[14] U. M. Sansom‐daly , C. E. Wakefield , C. Signorelli , et al., “Patterns and Predictors of Healthcare Use Among Adolescent and Young Adult Cancer Survivors Versus a Community Comparison Group,” Cancers 13, no. 21 (2021): 5270, 10.3390/cancers13215270. 34771435 PMC8582416

[15] J. K. N. Chan , C. U. Correll , C. S. M. Wong , et al., “Life Expectancy and Years of Potential Life Lost in People With Mental Disorders: A Systematic Review and Meta‐Analysis,” EClinicalMedicine 65 (2023): 102294, 10.1016/j.eclinm.2023.102294. 37965432 PMC10641487

[16] J. Y. Tan , G. Ge , C. E. Low , et al., “Suicide and Suicidal Ideation Among Survivors of Childhood Cancer,” JAMA Network Open 8, no. 2 (2025): e2457544, 10.1001/jamanetworkopen.2024.57544. 39960673 PMC11833522

[17] L. E. Frederiksen , F. Erdmann , L. Mader , et al., “Psychiatric Disorders in Childhood Cancer Survivors in Denmark, Finland, and Sweden: A Register‐Based Cohort Study From the SALiCCS Research Programme,” Lancet Psychiatry 9, no. 1 (2022): 35–45, 10.1016/s2215-0366(21)00387-4. 34822758

[18] T. Abdalla , D. B. Preen , J. D. Pole , et al., “Psychiatric Disorders in Childhood Cancer Survivors: A Retrospective Matched Cohort Study of Inpatient Hospitalisations and Community‐Based Mental Health Services Utilisation in Western Australia,” Australian and New Zealand Journal of Psychiatry 58, no. 6 (2024): 515–527, 10.1177/00048674241233871. 38404162 PMC11128143

[19] H. Forbes , H. Carreira , G. Funston , et al., “Early, Medium and Long‐Term Mental Health in Cancer Survivors Compared With Cancer‐Free Comparators: Matched Cohort Study Using Linked UK Electronic Health Records,” EClinicalMedicine 76 (2024): 102826, 10.1016/j.eclinm.2024.102826. 39318789 PMC11421364

[20] D. H. Brewster , D. Clark , L. Hopkins , et al., “Subsequent Hospitalisation Experience of 5‐Year Survivors of Childhood, Adolescent, and Young Adult Cancer in Scotland: A Population Based, Retrospective Cohort Study,” British Journal of Cancer 110, no. 5 (2014): 1342–1350, 10.1038/bjc.2013.788. 24366296 PMC3950849

