# Supplementary material for: Mental Health Outcomes Among Long‐Term Survivors of Childhood, Adolescent and Young Adult Cancer: A Scottish Population‐Based Cohort Study
Source: Psychooncology. 2026 Jun 21;35(6):e70529. doi: 10.1002/pon.70529 (PMC13283369; doi:10.1002/pon.70529)
Supplement: Supplementary file 1 — Supporting Information S1 [file PON-35-e70529-s001.docx]

**Supplementary material**

for

**Mental Health Outcomes Among Long-term Survivors of Childhood, Adolescent and Young Adult Cancer: a Scottish Population-Based Cohort Study**

Authors: Molinari E. et al.

**Figure S1. Flow diagram identifying source of data on first mental health event during follow-up.**


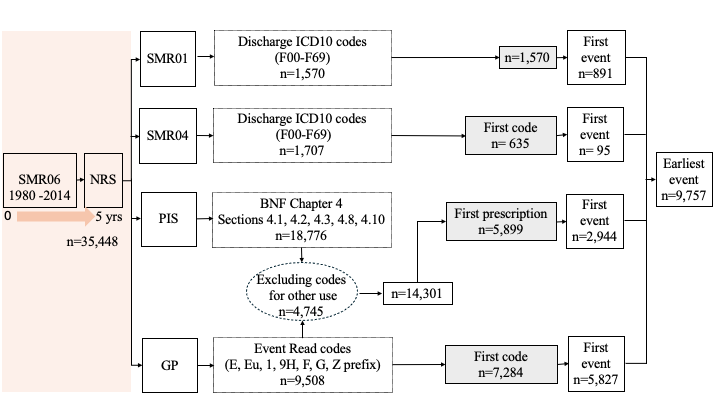


† The light orange box represents the cohort of cancer survivors alive at five years post-diagnosis (linked with NRS death records) together with their matched comparators (assigned a pseudo-date for cohort entry). The grey boxes show the counts of first mental health codes within each dataset separately SMR01, SMR 04, PIS, GP records, with “first code” referring to the earliest mental health code recorded per person within that dataset. “First event” refers to the earliest mental health contact per person across all datasets, after harmonising multiple codes and dates, resulting in the final counts of the earliest event.

‡ BNF, British National Formulary; GP, General Practitioner; MH, Mental Health; NRS, National Records of Scotland; PIS, Prescribing Information System; SMR, Scottish Morbidity Record.

**Figure S2. Kaplan–Meier estimates of cumulative probability of first mental health event among five-year cancer survivors and matched comparators.**

**
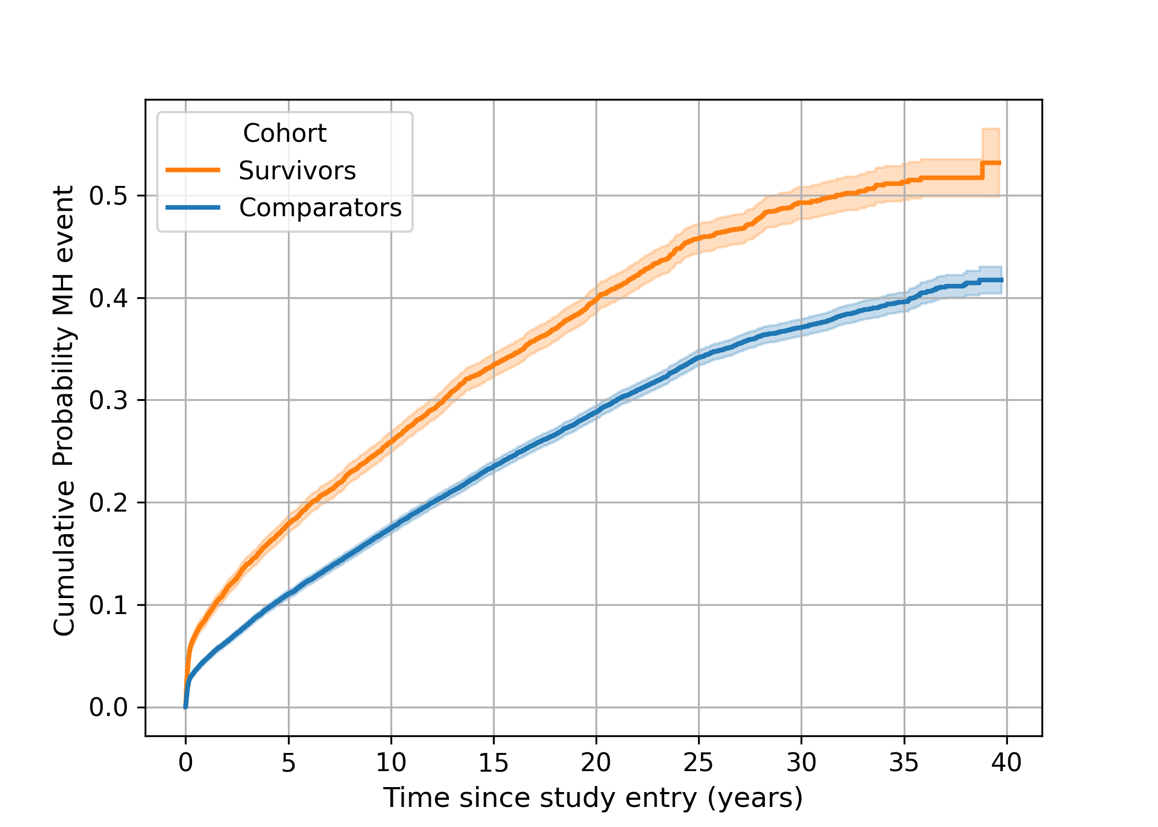
**

†Differences between groups were assessed using the log-rank test (p < 0.001).

**Table S.1. ICD-10 codes used to define cancer diagnoses.**

| **Category** | **ICD-10 codes** | **Description** |
| --- | --- | --- |
| Included | C00–C97 | Malignant neoplasms (invasive cancers) |
| Excluded | C77–C79 | Secondary malignant neoplasms |
| Excluded | D00–D09 | In situ neoplasms |
| Excluded | D10–D36 | Benign neoplasms |
| Excluded | D37–D48 | Neoplasms of uncertain or unknown behaviour |

† Cancer diagnoses were identified from the Scottish Cancer Registry (SMR06). Only invasive primary malignant neoplasms (ICD-10 C00–C97) were included. In situ, benign, and neoplasms of uncertain or unknown behaviour and secondary malignancies (C77-C79) were excluded. Classification was based on registry behaviour coding.

**Table S2. Diagnostic codes and prescribing definitions used to identify mental health outcomes.**

| **Data source** | **Category** | **Codes / Definition** |
| --- | --- | --- |
| **Included mental health diagnoses** | | |
| Hospital  (SMR01, SMR04) | Mental and behavioural disorders | ICD-10 F10–F48, F50–F99, F00–F09, F42-44–F49–F59, F60–F69 |
| Primary care  (Read codes) | Mental health diagnoses | E112., E113*, E02y300, 90v4., 8HHq.00, 146D.00, E115400, E11*, E110.11, Eu32*, Eu3*, Eu40*, Eu41*, F41*, E203100, E254300, E23.., E23xx, E24*, E244*, E25*, E26*, Eu145*, Eu114*, Eu1*, Eu2*, Eu22, Eu22y13, Eu25*, Eu33312, Eu32312, E101*, E10*, E1025*, E102500, E12z*, E121.00, E130*, E02*, E011*, E21*, 1B1A., 1V16.00, 1TDx, 1T3*, 9HC, Eu60*, Eu66x, Eu607, Eu0420 |
| Prescribing (PIS) | Drugs for anxiety and sleep disorders (anxiolytics, hypnotics) | BNF 4.1 |
|  | Antipsychotic drugs | BNF 4.2 |
|  | Antidepressant drugs | BNF 4.3 |
|  | Drugs used in substance dependence | BNF 4.8,4.10 |
|  |  |  |
| **Excluded non-mental indications** | | |
| Hospital  (SMR01, SMR04) | Developmental and learning disorders | ICD-10 F70–F79, F80–F89, F90–F98 |
| Primary care  (Read codes) | Neurological, developmental, and cognitive conditions | Eu81z00,1B1*, E3*, E31*, E2E0100, E2E*, 6A61.00, Eu90*, Eu9011, Eu90011, 9Ngp.00, 8BPT.00, E310.00, Eu70000, Eu72y00, Eu71100, F373.00, 9Ou3.00, E030400, dementia, learning disability, developmental disorders, behavioural problems, ADHD, autism |
| Prescribing (PIS) cross-referencing | Alternative non-psychiatric indications | Eu81z00, 1B1*, E3*, E31*, E2E0100, E2E*, 6A61.00, Eu90*, Eu9011, Eu90011, 9Ngp.00, 8BPT.00, E310.00, Eu70000, Eu72y00, Eu71100, F373.00, 9Ou3.00, E030400, F25., F251400, F254200, F254300, F258.00, F25y300, F25y400, F2A., F36yz00, F371200, F372.00, F374800, F37y100, F3y0.00, Fyu5900, N023., N035.12, 1B3A., E00., Eu06013, 9Of7.00, 667G.00, 667N.00, C108J12, C10920, C109211, C109B12, C10EB00. Pain, migraine, epilepsy, neuropathy, neuropathic pain, dementia, sleep disorders, insomnia, ADHD, autism |

† Mental health events were identified using hospital diagnoses (SMR01/SMR04), primary care diagnostic codes, and community prescribing data (PIS). Prescriptions were classified as mental health–related when consistent with sustained treatment and after excluding alternative non-psychiatric indications through diagnostic cross-referencing. To address the wide range of diagnostic and prescribing terminology, searches were performed using both code identifiers and descriptive terms (e.g., seizure, epilepsy, migraine, headache, pain, neuropathic pain, sleep disorder, dementia). When multiple eligible diagnostic or prescribing records were present, the earliest recorded event was used to define the outcome. Abbreviations: SMR, Scottish Morbidity Record; PIS, Prescribing Information System; BNF, British National Formulary.

**Table S3.** **Age at diagnosis, decade of diagnosis and type of cancer among five-year survivors of cancer diagnosed <40 years of age in Lothian 1980-2018**

| **Characteristic** | **Cancer survivors (>5 years)** |
| --- | --- |
| **Age at diagnosis, years (median [IQR])** |  |
| Children (0–9 years) | 3.87 [2.21–6.35] |
| Teenagers (10–24 years) | 20.50 [16.58–23.08] |
| Young adults (25–39 years) | 34.49 [30.63–37.52] |
| **Type of cancer, n (%)** |  |
| Melanomas and other skin | 2,095 (23.6) |
| Breast | 2,042 (23.0) |
| Germ cell and other gonadal | 1,162 (13.1) |
| Central nervous system (CNS, total) | 489 (5.5) |
| Grade I | 38 (0.4) |
| Grade II–IV | 451 (5.1) |
| Lymphomas (total) | 908 (8.3) |
| Hodgkin | 553 (6.2) |
| Non-Hodgkin | 355 (4.0) |
| Soft tissue / sarcomas | 174 (2.0) |
| Other carcinomas | 306 (3.5) |
| Others (unspecified) | 777 (8.8) |
| Renal | 146 (1.7) |
| Leukaemia (total) | 526 (5.9) |
| Acute lymphoblastic | 366 (4.1) |
| Acute myeloid | 131 (1.5) |
| Other leukaemia | 29 (0.3) |
| Bone | 117 (1.3) |
| Hepatic | 33 (0.4) |
| **Calendar period of diagnosis, n (%)** |  |
| 1980–1989 | 1,601 (18.1) |
| 1990–1999 | 2,120 (23.9) |
| 2000–2009 | 2,676 (30.2) |
| 2010–2018 | 2,386 (26.9) |

**Table S4.** Covariate-specific adjusted hazard ratios for first mental health event by follow-up interval (time-stratified Cox models).

| Covariate | Follow-up interval (years) | HR | 95% CI | p-value |
| --- | --- | --- | --- | --- |
| **Age** |  |  |  |  |
| Teenager vs Child | 0–1 | 1.21 | 0.67–2.20 | 0.52 |
| Teenager vs Child | >1–5 | 1.14 | 0.79–1.63 | 0.49 |
| Teenager vs Child | >5–10 | 1.11 | 0.73–1.69 | 0.62 |
| Teenager vs Child | >10–15 | 0.98 | 0.64–1.48 | 0.91 |
| Teenager vs Child | >15 | 1.04 | 0.71–1.52 | 0.85 |
| Young adult vs Child | 0–1 | 1.33 | 0.77–2.32 | 0.31 |
| Young adult vs Child | >1–5 | 1.26 | 0.91–1.75 | 0.16 |
| Young adult vs Child | >5–10 | 1.39 | 0.97–2.00 | 0.07 |
| Young adult vs Child | >10–15 | 1.08 | 0.75–1.54 | 0.69 |
| Young adult vs Child | >15 | 1.37 | 0.99–1.91 | 0.06 |
| **Decade of diagnosis** |  |  |  |  |
| 1990–1999 vs 1980–1989 | 0–1 | 1.79 | 1.18–2.72 | 0.006 |
| 1990–1999 vs 1980–1989 | >1–5 | 1.85 | 1.52–2.25 | <0.001 |
| 1990–1999 vs 1980–1989 | >5–10 | 2.91 | 2.50–3.38 | <0.001 |
| 1990–1999 vs 1980–1989 | >10–15 | 3.10 | 2.70–3.56 | <0.001 |
| 1990–1999 vs 1980–1989 | >15 | 0.55 | 0.49–0.62 | <0.001 |
| 2000–2009 vs 1980–1989 | 0–1 | 4.68 | 3.22–6.79 | <0.001 |
| 2000–2009 vs 1980–1989 | >1–5 | 4.72 | 3.96–5.62 | <0.001 |
| 2000–2009 vs 1980–1989 | >5–10 | 4.20 | 3.18–5.54 | <0.001 |
| 2000–2009 vs 1980–1989 | >10–15 | 2.15 | 1.06–4.18 | 0.04 |
| 2000–2009 vs 1980–1989 | >15 | 0.40 | 0.32–0.51 | <0.001 |
| 2010–2019 vs 1980–1989 | 0–1 | 4.54 | 3.14–6.57 | <0.001 |
| 2010–2019 vs 1980–1989 | >1–5 | 2.96 | 2.47–3.55 | <0.001 |
| 2010–2019 vs 1980–1989 | >5–10 | 1.46 | 1.18–1.80 | <0.001 |
| 2010–2019 vs 1980–1989 | >10–15 | 1.53 | 0.74–3.17 | 0.25 |
| 2010–2019 vs 1980–1989 | >15 | 0.32 | 0.09–1.16 | 0.08 |

**Supplementary Methods**

**Statistical environment, software, and packages**

All analyses were conducted within the secure DataLoch Trusted Research Environment using Python (v3.11) for data preparation, descriptive analyses, and model fitting. The DataLoch Advanced Analytic Workbench (AAW) operated on Linux (Ubuntu 20.04) and provided a controlled Jupyter Notebook interface with restricted package installation and no external connectivity, which informed both the analytic design and library selection. Key open-source libraries included pandas, numpy, scipy, matplotlib, lifelines, statsmodels, and scikit-survival.

**Matching**

The DataLoch team performed cohort matching prior to data release using the MatchIt package in R (version 4.5.0). Nearest-neighbour matching without replacement (ratio 3:1) was conducted on year of birth, sex, and quintile of the Scottish Index of Multiple Deprivation (SIMD). Matched comparators were assigned the diagnosis date of their paired survivor as a pseudo-diagnosis date to ensure alignment of follow-up.

**Mental health event identification.**

MH outcomes were defined using multiple sources: International Classification of Diseases (ICD)-10 codes (F00–F69) in hospital discharge records (SMR01/SMR04), Read codes for MH symptoms or diagnoses in GP records, and psychotropic medication dispensing in PIS (British National Formulary, BNF, Chapters 4.1, 4.2, 4.3, 4.8, 4.10: antidepressants, anxiolytics, hypnotics, antipsychotics, and anti-seizure medications). Full diagnostic codes are provided in Supplementary Table S.2. Dispensed prescriptions were included as valid proxies for a MH event only if dispensed for six months or more. Prescriptions associated with non-mental health indications (e.g., epilepsy, migraine, pain, neuropathic pain, sleep disorders, or dementia) were excluded based on diagnostic information available in primary care records. Full cross-referencing codes are given in Supplementary Table S.2. Multiple diagnostic or prescribing codes could occur within and across data sources. Within each dataset, the earliest eligible mental health event per individual was identified. The primary outcome was defined as the first mental health event across all data sources, determined by the earliest recorded date after harmonisation of multiple records. Individuals with any such record occurring before cancer diagnosis (or pseudo-diagnosis for comparators) were classified as having prior mental health history. Mental health events occurring between diagnosis and study entry (five years post-diagnosis) were not included, as the study was designed to evaluate incident mental health outcomes arising during long-term survivorship. Supplementary Figure S1 illustrates the identification of first mental health codes within each data source and the derivation of the earliest event across sources.

**Statistical analysis (additional details)**

Follow-up began at cohort entry (five years after diagnosis or pseudo-diagnosis for comparators). Individuals were followed until the earliest of first mental health event, death, or end of study follow-up, whichever occurred first. Individuals who died out of Scotland (n = 3) and their matched comparators were excluded from the analysis. Time since cohort entry was used as the time scale.

Mental health events occurring between cancer diagnosis and cohort entry were not included in the outcome definition, as the analysis focused on incident mental health outcomes arising during long-term survivorship rather than during the early post-diagnosis period.

To account for the matched design, Cox models were fitted with clustering by matched set to allow for within-set correlation and obtain robust standard errors. Where models included sparse categories and convergence warnings occurred, a small ridge (L2) penalisation was applied to improve numerical stability. This did not materially affect effect estimates. Model assumptions were evaluated using standard diagnostic methods. Multicollinearity was assessed using variance inflation factors (VIF), with values <5 considered acceptable. Pairwise correlations between covariates were examined and were all <0.80.
